# De Novo mutation in Epidermal growth factor receptor (EGFR)-D761Y responding to third generation tyrosine kinase inhibitor Osimertinib: A case report

**DOI:** 10.1097/MD.0000000000029332

**Published:** 2022-07-22

**Authors:** Yingying Ding, Hongliang Dong, YongCheng Li, Lei Liu, Ying Cai, Ying Wang, Shengya Tian, Chengtao Dai

**Affiliations:** a Department of Respiratory and Critical Care Medicine, The First People's Hospital of Xiaoshan District, Xiaoshan Affiliated Hospital of Wenzhou Medical University, Hangzhou, 311200, Zhejiang, China; b Department of Critical Care Medicine, The First People's Hospital of Xiaoshan District, Xiaoshan Affiliated Hospital of Wenzhou Medical University, Hangzhou, 311200, Zhejiang, China; c Amoy Diagnostics Co Ltd., 39 Dingshan Road, Haicang District Xiamen, Fujian, CN 361027.

**Keywords:** case report, EGFR-D761Y mutation, EGFR-L858R, NSCLC, Osimertinib

## Abstract

Non-small cell lung cancer (NSCLC) is the most common type of lung cancer. Epidermal growth factor receptor (EGFR) mutations are the most common accurate gene targets. However, the lack of case reports or cohort studies on the exceptionally rare mutations limit the acquisition of deeper insights.

**Patient concerns:**

A 76-year-old female nonsmoker presented to our hospital with a one-week disease history of cough accompanied by shortness of breath.

**Diagnosis:**

Contrast-enhanced CT scan showed right pleural effusion with scattered inflammation and consolidation in the right upper lung. Tumor marker display showed obvious increased. Histopathology of the pulmonary mass combined with Immunohistochemical staining indicated lung adenocarcinoma. Contrast-enhanced magnetic resonance imaging suggested brain metastases. ECT scan showed bone metastasis. The patient was thus diagnosed as right lung adenocarcinoma of stage IV (cT3N3M1c). Next generation sequencing was performed to profile the mutation status of known oncogenic driver mutations, and only EGFR-D761Y in exon 19 (allelic frequency, AF: 0.53%) mutation was found.

**Interventions:**

The patient was accordingly treated with the third generation EGFR-Epidermal growth factor receptor tyrosine kinase inhibitor (TKI) Osimertinib (80 mg, qd). Accompanied with whole brain radiotherapy (DT3000c Gy/10f) for brain metastases, technetium methylene diphosphonate injection was performed for bone metastases.

**Outcomes:**

The efficacy of the first-line Osimertinib treatment for 1 month was assessed as PR per RECIST version 1.1. The NSCLC patient harboring EGFR-D761Y mutation detected prior to the EGFR L858R mutation was benefited from the third-generation EGFR-TKI Osimertinib and had a worse prognosis than with other EGFR mutations according to data from previous case reports.

**Conclusions:**

This case reported a NSCLC patient with de novo mutation of EGFR-D761Y responding to third generation TKI Osimertinib.

## 1. Introduction

Lung cancer is a globally malignant tumor with high mortality. Non-small cell lung cancer (NSCLC) is the most common type of lung cancer, accounting for 80% and 85% of lung cancer cases.^[1]^ The standard treatment of advanced NSCLC has been revolutionized by the development of accurate gene targets.^[2,3]^ Epidermal growth factor receptor (EGFR) mutations are the most common accurate gene targets.^[4]^ The application of EGFR-Epidermal growth factor receptor tyrosine kinase inhibitors (TKIs) in patients harboring EGFR mutation has been a representative model of precise medicine in the treatment of advanced NSCLC.^[5]^ However, majority studies only included patients with common EGFR mutation sites which comprise approximately 90% of EGFR mutations, such as deletion in exon 19 or L858R point mutation in exon 21.^[6,7]^ The remaining 10% uncommon mutation sites make up a heterogeneous group of single (i.e., G719X, S768I, L861Q) and compound (i.e., G719X+L858R, L861Q+L858 M, G719X+S768I) gene alterations within exons 18 to 21 which exhibiting differential responses to EGFR inhibitors.^[8,9]^

Here, we report a NSCLC patient harboring de novo EGFR-D761Y mutation and benefited from the third-generation EGFR-TKI Osimertinib. We also performed a literature review on the clinical characteristics of patients with D761Y mutation together with a descriptive analysis about their response to EGFR-TKI according to the data from previous case reports.

## 2. Ethics and Methods

Written informed consent was obtained from the individual and approval of the Institutional Medical Ethics Committee of XiaoShan First People's Hospital, HangZhou.

Genomic DNA was extracted from the peripheral blood samples of the patient. The AmoyDx Essential next generation sequencing (NGS) panel (Illumina NovaSeq 6000/Nestseq 500 platform, Inc., AmoyDx, China) and 448 gene panel were used for qualitative detection of single nucleotide variants, copy number variants, indels, and gene rearrangements in the 10 genes lung panel (including EGFR, KRAS, RET, ROS1, ALK, NRAS, BRAF, MET, PIK3CA, and HER2) in NSCLC in accordance with the manufacturer's instructions. Each result was qualified only when the sample coverage was more than 98%, the average original depth (Mean Depth) was more than 10,000× and the average effective depth (SSBCDepth) was more than 500×. The threshold of mutation frequency for mutation was 0.4%. The mutant copy number (DSBCDepth) was not less than 2.

PD-L1 positivity was measured by Immunohistochemical staining on formalin-fixed paraffin-embedded (FFPE) specimens.

## 3. Case presentation

**Figure 1.**
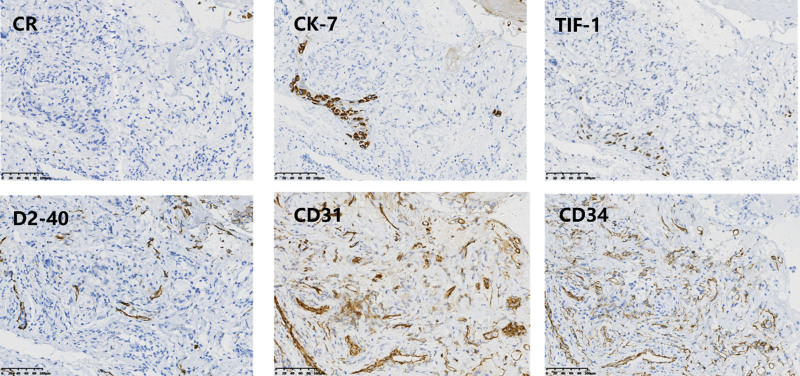
Immunohistochemistry for CR, CK7, TIF-1, D2–40, CD31, CD34, of the specimen, which showed lung adenocarcinoma.10X.

A 76-year-old female non-smoker presented to our hospital with a one-week disease history of cough accompanied by shortness of breath. Contrast-enhanced computed tomography (CT) scan showed right pleural effusion with scattered inflammation and consolidation in the right upper lung. Tumor marker display: Ferritin 445.23 ng/mL, CA 125 192.00KU/L, carcinoembryonic antigen 4.61 ug/L, Cytokeratin 19 fragment (CYFRA 21–1) 10.56 ng/mL. Histopathology of the pulmonary mass combined with immunohistochemistry showed in Figure 1 indicated lung adenocarcinoma: CR (-), CK7(+), TTF-1 (+), D2–40 (-), CD31 (vascular+), CD34 (vascular+). Contrast-enhanced magnetic resonance imaging showed multiple space-occupying lesions, which were considered as brain metastases. Besides, Emission computed tomography scan showed bone metastasis. The patient was thus diagnosed as right lung adenocarcinoma of stage IV (cT3N3M1c). NGS was performed on thoracoscopic biopsy specimens to profile the mutation status of 10 genes associated with known oncogenic driver mutations (EGFR, ALK, HER2, BRAF, MET, ROS1, RET, NRAS, KRAS and PIK3CA) in NSCLC. Only 1 mutation was found, namely EGFR-D761Y in exon 19 (allelic frequency, AF: 0.53%). And the results showed PD-L1 was positive, TPS:10%. The patient was accordingly treated with the third generation EGFR-TKI Osimertinib (80 mg, qd). Accompanied with whole brain radiotherapy (DT3000c Gy/10f) for brain metastases, technetium methylene diphosphonate injection was performed for bone metastases.

**Figure 2.**
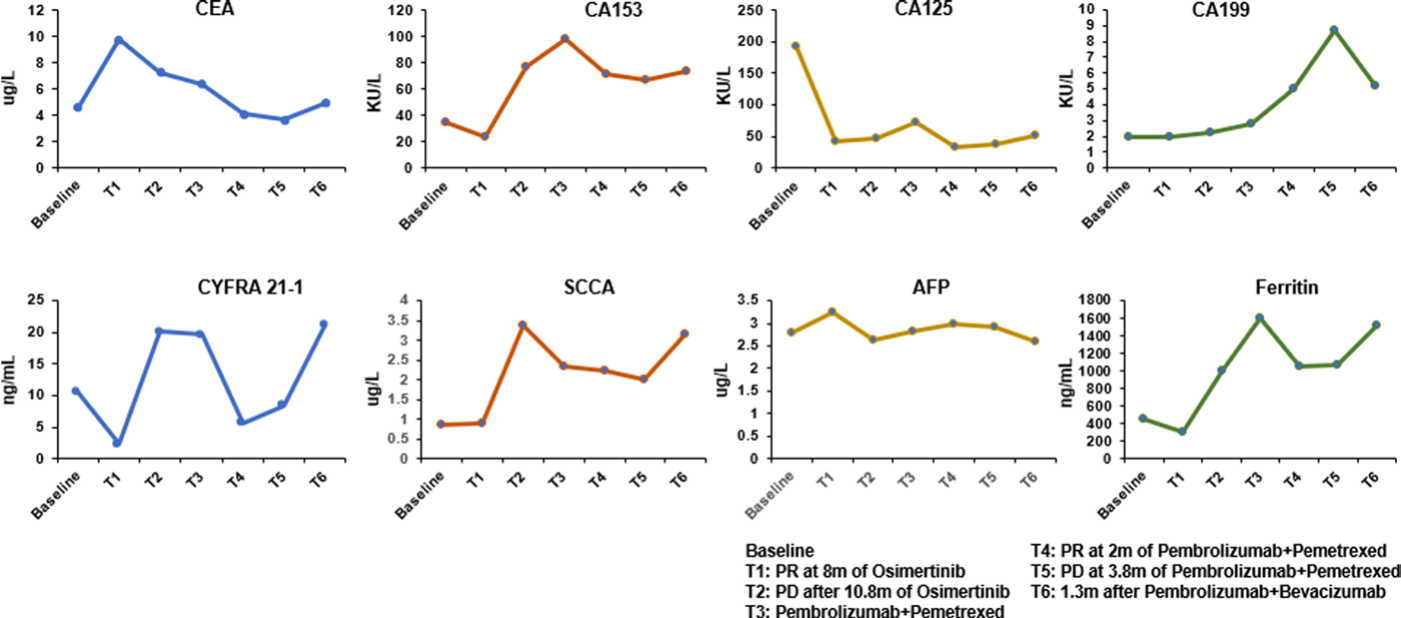
Representative tumor marker at indicated time points, shown along with corresponding treatment regimen and efficacy evaluation.

Following up contrast-enhanced CT scans (1 month later, 3 months later, and 8 months later) among 8 months revealed obvious decrease of primary lesion and mediastinal lymph nodes compared with previous images, the same significantly decrease of brain metastases lesion (the minimum diameter of the target nodule: 6.4 cm vs 4.0 cm; the longest diameter: 12.7 cm vs 7.7 cm). The efficacy of the first-line Osimertinib treatment for 1 month was assessed as partial response (PR) per RECIST version 1.1. At this time, Ferritin, CA125 and carcinoembryonic antigen indexes were significantly decreased, but CYFRA 21 to 1 indexes were increased (Fig. 2). The efficacy of the Osimertinib treatment was still PR after 3 months, and was stable disease (SD) after 8 months.

**Figure 3.**
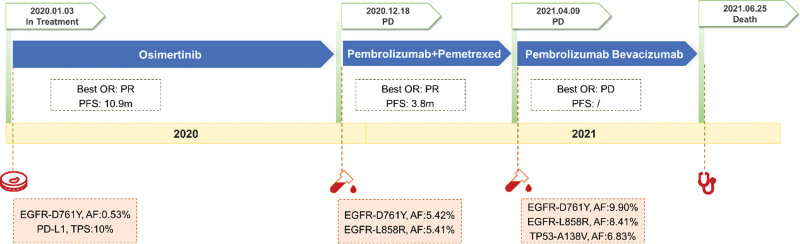
Diagram of the course of disease management, showing different treatment regimens prescribed and the results of mutation analyses.

After continued Osimertinib administration for 10 months, the patient developed severe facial swelling. Chest contrast-enhanced CT scans revealed significant enlargement of the lesion in the right lung involving hilum and pulmonary artery, sporadic nodules in the left lung, and accompanied with superior vena cava occlusion, which led to a reevaluation of progressive disease (PD). There was a substantial intrahepatic mass at the left liver to indicated liver metastasis. The progression-free survival (PFS) of first-line Osimertinib was therefore 10.9 month (Fig. 3). Then NGS was performed on the plasma ctDNA to profile the mutation status of 10 NSCLC-related target genes. In addition to the EGFR-D761Y mutation detected in the previous sequencing tests, the AF was increased to 5.25% (D761Y, AF: 5.42%), a mutation of EGFR-L858R in exon 21 was identified (L858R, AF: 5.41%).

Based on PD-L1 positive, immunotherapy with pembrolizumab (100 mg, q3w) combine chemotherapy with pemetrexed (400 mg, q3w) was prescribed as second-line therapy. The patient was received 4 cycles of immunotherapy plus chemotherapy, follow-up 2 cycles of mono-immunotherapy maintenance. The efficacy of the second-line immunotherapy and chemotherapy treatment was assessed as partial response (PR) among the immunotherapy plus chemotherapy treatment. Contrast-enhanced CT showed size of the lesion was significantly reduced.

However, it led to a reevaluation of progressive disease on the follow-up 2 cycles of mono-immunotherapy maintenance after 3.8 months, as chest contrast-enhanced CT scans revealed a significant enlargement of the lesion in the right lung and contrast-enhanced magnetic resonance imaging suggested progress to brain metastases. The plasma ctDNA were subjected to capture-based ultra-deep sequencing that target the exons and critical introns of 448 cancer-related genes (Amoy Diagnostics, Xiamen, China). In addition to the 2 EGFR mutations detected the previous sequencing tests (L858R, AF: 8.41%; D761Y, AF:9.9%), a mutation in TP53 A138 V was identified (AF: 6.83%). Tumor marker display the indexes of ferritin, CA153, CA125, CYFRA 21–1 and squamous cell carcinoma antigen (SCCAg) were significantly increased, especially the ferritin indexes was as high as 1061.18 ng/mL.

**Figure 4.**
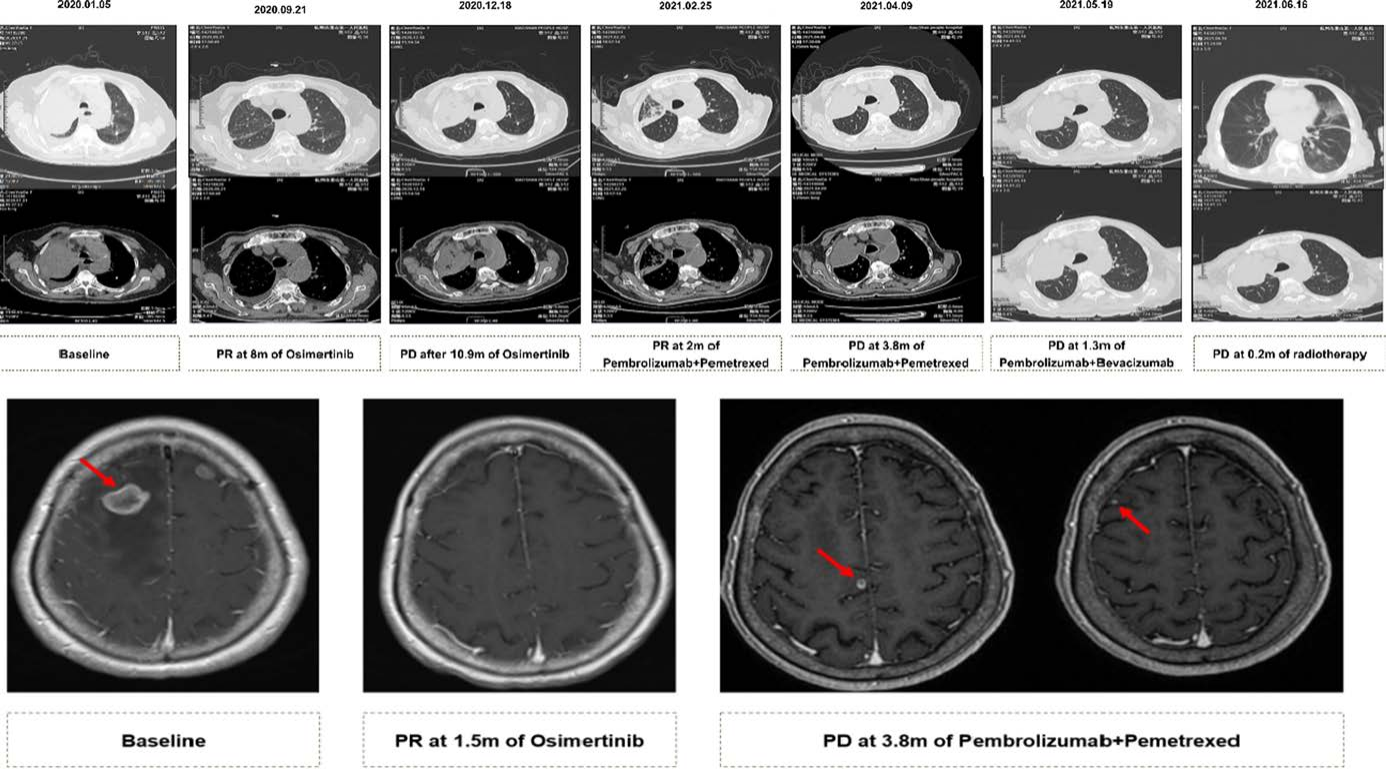
Representative CT images and MRI images at indicated time points, shown along with corresponding treatment regimen and efficacy evaluation.

After the disease progressed, the immunotherapy plus chemotherapy regimen was replaced with immunotherapy plus anti-vascular therapy: the pembrolizumab (100 mg, q3w) plus bevacizumab (300 mg, q3w) for the treatment of lung cancer. Then, PET-CT showed metastases in the 8th, 10th, 11th thoracic vertebrae and the 1st, 3rd, 4th, and 5th lumbar vertebrae, and lumbar radiotherapy (T7–8 DT3000cGy/10f, 300cGY/f, 1f/d, 5f/w) was performed. (Figs. 3 and 4). Among 1.3 months of treatment, the radio graphic assessment was still progressive disease, and the tumor markers were still increasing. There were no significant adverse events throughout the course of treatment. After failing to respond to salvage treatment, the patient died on 2.5 months later. An informed consent was obtained for the publication of the case report.

## 4. Discussion

This case reported a NSCLC patient with EGFR-D761Y mutation detected priors to the EGFR L858R mutation, who benefited from the third-generation EGFR-TKI Osimertinib. The PFS with Osimertinib treatment is 10.9 month.

D761Y is an extremely rare mutation of EGFR in exon19, with few cases reported before. In 2006, Tokumo et al reported a progressed-death NSCLC patient with EGFR L858R/D761Y mutation, which was clinically reported to be resistance to Gefitinib treatment.^[10]^ Subsequently, Balak et al reported that the EGFR D761Y mutation was a “novel secondary mutation” that appeared in a metastatic brain lesion after the acquisition of Gefitinib resistance published in clinical cancer research. Based on this case, D761Y is widely defined to be a secondary drug-resistant mutation of EGFR-TKI.^[11]^ However, in the following year, Tokumo et al wrote a letter to the editor of clinical cancer research, to raise doubts about the case report of Balak et al, which was unable to evaluate the D761Y mutation in the pretreatment specimen because of no remaining DNA. Thus, it was unclear whether the D761Y mutation was “inherent” or “secondary” to gefitinib treatment.^[12]^ Until 2020, Zhu et al reported the clinical efficacy of Icotinib and Osimertinib in a patient with metastatic NSCLC carryingan EGFR L858R/D761Y co-mutation. A 62-year-old non-smoking woman with advanced lung adenocarcinoma who responded with stable disease (SD) to sequential treatments with Icotinib and Osimertinib, the PFS with Osimertinib treatment is much longer than that with Icotinib (19 months vs 8.2 months) and the OS is more than 3 years.^[13]^ Besides, an in vitro study has showed that despite displaying resistance to first-generation reversible EGFR-TKIs, L858R/D761Y mutant cells were sensitive to irreversible inhibitors, especially the third-generation EGFR-TKIs.^[14]^

However, in this case, we reported a lung adenocarcinoma patient carrying D761Y mutation present before EGFR-TKI treatment. NGS was used to detect the large specimens of the patient's thoracoscopy and the results observed D761Y mutation (AF: 0.53%) as well as PD-L1 positive (10%). Then the L858R mutation (AF: 5.41%) /D761Y mutation (AF: 5.42%) compound mutation was detected after treated with Osimertinib for about 8 months. This is the first report that supported the D761Y mutation was present before EGFR-TKI treatment, which indicating that this mutation was a de novo mutation. The third-generation EGFR-TKI of Osimertinib is clinically effective with a large PR in the treatment target D761Y, which is better than the SD reported by Zhu et al, indicating that the D761Y mutation has a poorer prognosis than other EGFR mutations.

Immunotherapy (IO) in combination with chemotherapy has been an effective therapeutic selection to control tumor and prolong the overall survival (OS) of advanced NSCLC patients. It has been reported that NSCLC patients with EGFR mutation may possibly benefit from combined immunotherapy.^[15]^ But a significant proportion of patients still suffer from disease progression.^[16]^ In our case, the efficacy of the second-line immunotherapy combined with chemotherapy treatment was assessed as PR after 3 months before disease progression. It has been reported that the response to prior EGFR-TKI was associated with the outcomes of subsequent immunotherapy remains: for NSCLC patients with T790 M mutation, patients with short TKI-PFS conferred better response to immunotherapy than those with long.^[17]^ Thus, the PFS is as short as about 3 months after second-line immunotherapy combined with chemotherapy treatment, possibly suggesting that patient carrying D761Y mutation not only present with a poorer prognosis than other EGFR mutations but also has association with the outcome of subsequent immunotherapy. Although it has been reported that patients with EGFR-mutated NSCLC could achieve clinical benefits of immune checkpoint inhibitors (ICIs) agents, the unique characteristics of TME of EGFR-mutated NSCLC such as metabolism, general immune status, immune cell infiltration, cytokines and soluble molecules affect the efficacy of ICIs in the patients with EGFR-mutated NSCLC. Underlying mechanisms need to be further explored.

## Author contributions

Conceptualization: Hongliang Dong, Yingying Ding.

Data curation: Ying Cai, Yingying Ding.

Formal analysis: Lei Liu, Yingying Ding.

Funding acquisition: Hongliang Dong.

Investigation: Yingying Ding.

Methodology: Yingying Ding.

Project administration: Shengya Tian, Yingying Ding.

Resources: Yingying Ding, Yongcheng Li.

Software: Chengtao Dai.

Supervision: Yingying Ding.

Validation: Yingying Ding.

Visualization: Yingying Ding.

Writing – original draft: Ying Wang.

Writing – review & editing: Shengya Tian, Ying Wang.
